# Effect of Focal Laser Photocoagulation on the Ganglion Cell Complex Thickness in Acute Central Serous Chorioretinopathy

**DOI:** 10.3390/jcm13041064

**Published:** 2024-02-13

**Authors:** A Young Lee, Jinyoung Choi, Sang Un Lee, Chul Woo Kim, Daniel Duck-Jin Hwang

**Affiliations:** 1 Department of Ophthalmology, Hangil Eye Hospital, Incheon 21388, Republic of Korea; 2 Department of Ophthalmology, Catholic Kwandong University College of Medicine, Incheon 22711, Republic of Korea

**Keywords:** central serous chorioretinopathy, optical coherence tomography, ganglion cell complex

## Abstract

This study investigated the changes in the ganglion cell complex (GCC) of patients with acute central serous chorioretinopathy (CSC) following focal laser photocoagulation (FLP) and sought to determine its correlation with visual acuity (VA). Our retrospective study was conducted on 30 patients diagnosed with acute CSC between January 2015 and April 2022, who underwent FLP within 3 months of symptom onset. The study assessed GCC changes by measuring the thickness of its inner retinal layers—retinal nerve fiber layer (RNFL), ganglion cell layer (GCL), and inner plexiform layer (IPL) using optical coherence tomography (OCT). GCC thickness was compared between the affected and unaffected eyes and a healthy control group. VA was also assessed at baseline and at 1, 3, and 6 months post-treatment. VA showed significant improvement from 0.20 ± 0.14 at baseline to 0.10 ± 0.12 logMAR at 6 months post-treatment (*p* = 0.003). There was no significant change in GCC thickness over the 6-month period. No significant differences in GCC thickness were observed when comparing CSC eyes with fellow eyes or with normal controls at any time point. Final VA was significantly related only to baseline VA, with no correlation found with other factors, including RNFL, GCL, and IPL thickness. In summary, for patients with acute CSC undergoing FLP, our findings indicate that there is no significant change in GCC thickness detectable by OCT before and after the resolution of subretinal fluid (SRF), despite improvements in VA post-laser treatment. This suggests that any potential impact of FLP on GCC thickness may be minimal and not discernible with the current measurement methods, such as OCT, emphasizing that VA improvements may be primarily associated with alterations in the outer retina rather than the inner retina. Further studies with extended follow-up durations are warranted to evaluate any potential long-term changes in GCC.

## 1. Introduction

Central serous chorioretinopathy (CSC) is linked to abnormalities in the retinal pigment epithelium (RPE), hyperpermeability, and changes in the choroid, which is characterized by a serous detachment of the neurosensory retina at the posterior pole [1,2]. Quantitative analysis is now possible because of the development of optical coherence tomography (OCT) devices, making it possible to learn more about the diagnosis and progression of ocular diseases. Since CSC is categorized as a pachychoroid spectrum disorder, most investigations to date have been on the outer retina and choroid [3,4,5], and just a few studies have been conducted on the inner retina [4,6,7,8,9]. 

The three layers in the inner retinal layers—retinal nerve fiber layer (RNFL), ganglion cell layer (GCL), and inner plexiform layer (IPL)—combine to form the ganglion cell complex (GCC). These layers comprise the ganglion cell’s axon, cell body, and dendrite, respectively. Many studies have shown the significance of GCC in glaucoma and diseases of the optic nerve [10,11]; furthermore, inflammatory, ischemic or retinal degenerative conditions have been reported to cause GCC abnormalities [12,13,14,15,16]. A prior study showed that the GCL and RNFL thickness reduced in acute CSC as the subretinal fluid (SRF) decreased [4]; in addition, other investigations showed that the GCC thickness decreased in comparison to normal [6] or fellow eyes [9] when SRF was present. However, there have been recent reports reporting conflicting results that there is no difference in GCC thickness between resolved acute CSC eyes and normal eyes [7,8]. Additionally, to our knowledge, there has been no study that has investigated changes in GCC only in laser-treated patients with acute CSC.

In this study, we aimed to investigate (1) whether there was any significant difference in the thickness of the GCC when compared to the unaffected eye or a healthy control group, and (2) whether focal laser photocoagulation influences the thickness of GCC by measuring it before and after the complete resolution of SRF. Additionally, we examined whether there is a correlation between GCC thickness and best-corrected visual acuity (VA).

## 2. Materials and Methods

This retrospective study was conducted in accordance with the principles of the Declaration of Helsinki. The Institutional Review Board (IRB) of Hangil Eye Hospital approved this study (IRB no. 23002) and waived the requirement for informed consent from the study participants owing to the retrospective nature of the study.

### 2.1. Patients

**Figure 1:**
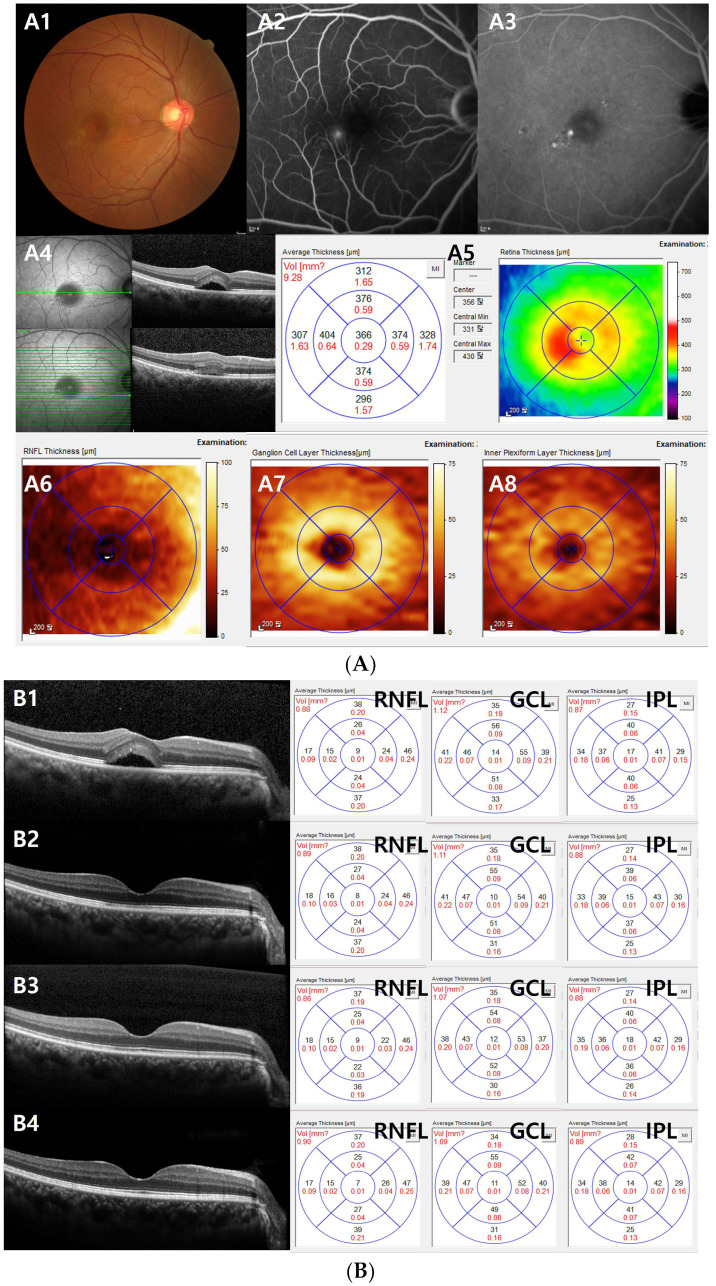
(**A**) Representative case of a 48-year-old male patient with acute central serous chorioretinopathy (CSC). (**A1**) Color fundus photography, (**A2**) fluorescein angiography (FA), (**A3**) indocyanine green angiography, (**A4**) infrared (IR) and optical coherence tomography (OCT) images, (**A5**) average thickness and volume of macula based on ETDRS circles, (**A6**) retinal nerve fiber layer (RNFL) thickness map, (**A7**) ganglion cell layer (GCL) thickness map, (**A8**) inner plexiform layer (IPL) thickness map. FA (**A2**) shows leakage of dye in ink blot pattern and OCT. (**A4**) shows increased subfoveal choroidal thickness and the presence of subretinal fluid. IR (**A4**) showed decreased reflectance on foveal center due to SRF. (**B**) This is the same case for (**A**). (**B1**–**B4**) Changes in the ganglion cell complex thickness after focal laser over time. With the gradual disappearance of subretinal fluid over time, RNFL, GCL, and IPL thickness remained unchanged. (**B1**) First visit, (**B2**) 1 month follow-up, (**B3**) 3 months follow-up, and (**B4**) 6 months follow-up after focal laser photocoagulation.

Among the patients who visited the hospital from January 2015 to April 2022, patients with acute CSC were included consecutively. The inclusion criteria were (1) patients with first-onset acute CSC, (2) who underwent focal laser photocoagulation within 3 months of symptom onset, and (3) SRF lasting no longer than 6 months on an onset basis (Figure 1). The exclusion criteria included (1) secondary CSC associated with systemic disease or medication history; (2) the presence of other potentially conflicting retinal pathologies, such as age-related macular degeneration (AMD), polypoidal choroidal vasculopathy (PCV), pachychoroid neovasculopathy (PNV), and pachychoroid pigment epitheliopathy (PPE); (3) patients who underwent intravitreal injection or photodynamic therapy for CSC treatment; (4) patients with glaucoma and optic nerve disease in either eye; and (5) patients who underwent retinal surgery. Age- and gender-matched groups were created for comparison with normal eyes. The control group was randomly selected according to age and gender from our hospital’s database of healthy control groups.

Only one ophthalmologist (DDH) performed all focal laser photocoagulation. The focal laser parameters used were an argon green laser (wavelength 532 nm), 100 μm spot size, <200 mW power, and <100 ms duration. Direct focal laser photocoagulation to juxtafoveal and extrafoveal leaks was applied to produce a minimally visible laser burn at the fluorescein angiogram-guided leakage, while taking care not to disrupt areas of retinal pigment epithelium detachment.

### 2.2. Ophthalmic Examination 

All CSC cases were diagnosed by means of fundus examinations, fluorescein angiography (FA), indocyanine green angiography (ICGA), and OCT images by one retinal specialist (DDH). A confocal scanning laser ophthalmoscope (Heidelberg Retina Angiograph, HRA; Heidelberg Engineering, Heidelberg, Germany) was used to perform simultaneous FA and ICGA on all CSC cases. The diagnosis of acute CSC was made by observing the existence of serous detachment of the neurosensory retina that affects the macula, as shown by OCT. Additionally, there was evidence of leaking at the level of the RPE on FA. Only classic acute CSC with a symptom duration of less than 3 months since the first episode was included in the acute CSC group. On the other hand, based on the Daruich and colleagues’ classification scheme [2], chronic CSC was excluded and any other diseases such as neovascular AMD, PCV, PNV, PPE, or CSC complicated by the choroidal neovascularization also were excluded using fundus examinations, FA, and ICGA. In some patients, OCT angiography (Swept source OCT, PLEX Elite™ 9000, Carl Zeiss Meditec, Jena, Germany) was also performed to accurately diagnose the disease and only acute CSC cases were selected as our study cases. Best-corrected VA (logMAR), intraocular pressure (NT-530P, Nidek, Aichi, Japan), post-mydriatic fundus photo (Optos PLC, Dunfermline, Scotland, UK), and SD-OCT (The Spectralis HRA ± OCT Version 6.9a; Heidelberg Engineering, Heidelberg, Germany) were performed in all the patients at the first visit and at 1, 3, and 6 months after focal laser photocoagulation.

### 2.3. OCT

A macula scan was performed with a 30° × 20° cube with 25 raster lines separated by 234 μm, each containing 768 pixels. Central macular thickness (CMT) was defined as the average thickness inside the 1 mm circle of the Early Treatment Diabetic Retinopathy Study (ETDRS) map. All 10 layers of the retina can be detected using the automatic segmentation algorithm of Heidelberg Eye Explorer software, of which the top 3 layers, RNFL, GCL, and IPL, are defined as GCC. If segmentation errors occurred after automatic segmentation, the authors (AYL and JC) manually adjusted the lines representing the RNFL, GCL, and IPL in each of the 25 lines. To minimize measurement error, we did not use the central 1 mm measurement value for RNFL, GCL, and IPL in this study. Instead, the average thickness of the 6 mm circle was calculated by dividing the volume value of the ETDRS 6 mm circle provided by OCT by 9π and then multiplying by 1000 (the average thickness of the 6 mm circle = volume of the 6 mm circle ÷ 9 π × 1000). Additionally, we analyzed the change in the average GCC thickness of sectors with leakage. For example, if there was leakage in the outer nasal sector, the change in the average GCC thickness of the outer nasal sector was investigated.

### 2.4. Statistical Analysis

Statistical analysis was performed using SPSS software (version 25.0; SPSS Inc., Chicago, IL, USA). A paired *t*-test was used for comparison with the fellow eye, and an independent *t*-test was used for comparison with the normal eye. Temporal changes in the study eye were analyzed using paired *t*-tests. The analysis of variance (ANOVA) test was adopted for variables using repeated measures. A multiple regression model was used to evaluate whether there were factors affecting the final VA. Baseline VA, age, sex, RNFL, GCL, and IPL thickness at baseline and at 6 months were corrected. A *p*-value of less than 0.05 was defined as significant.

## 3. Results

A total of 30 patients (30 eyes) with acute CSC were included in the study. Their average age was 54.21 ± 9.72 years; there were 21 males and 9 females. No significant differences were observed in the gender and age between the CSC group and the normal control group (Table 1).

### 3.1. Changes in the Best-Corrected Visual Acuity, Intraocular Pressure (IOP), and CMT

The average VA at the first visit was 0.20 ± 0.14 logMAR, and the final VA was 0.10 ± 0.12 logMAR, demonstrating a significant improvement (*p* = 0.003). The IOP was 14.60 ± 2.57 mmHg at the first visit and 15.82 ± 2.40 at 6 months, showing no significant difference (*p* = 0.459). CMT decreased significantly from 423.90 ± 118.62 µm initially to 255.28 ± 63.93 µm at 6 months (*p* < 0.001).

### 3.2. Changes in the GCC Thickness (RNFL, GCL, and IPL)

At the initial visit, no significant differences were observed in GCC thickness upon comparing CSC eyes to fellow eyes, CSC eyes to normal controls, and fellow eyes to normal controls (Table 2). In the CSC eyes, no significant difference was observed between the initial, 1, 3, and 6 month values of GCC (Table 3). Upon comparing the RNFL, GCL, and IPL thickness between the baseline and follow-up for each value, no significant differences were observed at any time point from the first visit to the 6 month follow-up visit after focal laser photocoagulation.

Additionally, we analyzed the change in the average GCC thickness of sectors with leakage. Upon comparing the GCC thickness between the baseline and follow-up for each value, no significant differences were observed at any time point from the first visit to the 6 month follow-up visit after focal laser photocoagulation (Table 4).

### 3.3. Factors Related to Final Visual Acuity

A correlation analysis was performed to determine the factors affecting the final VA. Final VA had a significant relationship only with baseline VA, whereas other factors, including age, sex, RNFL, GCL, and IPL thickness at the first visit and at 6 months after focal laser photocoagulation, were not significantly related (Table 5). 

## 4. Discussion

This study aimed to analyze the change in the GCC of patients with acute CSC who had undergone focal laser photocoagulation. When SRF was observed at baseline, there was no significant difference in GCC thickness compared to the fellow or healthy control eyes. Six months follow-up after focal laser photocoagulation with no SRF, the GCC thickness displayed no noteworthy alterations compared to baseline, with no significant differences observed in RNFL, GCL, or IPL thicknesses.

Although numerous factors have been associated with CSC, RPE dysfunction and choroidal changes have been proven to be the principal contributing elements [17]. Consequently, most CSC research has concentrated on the outer retina, with inner retina studies being relatively scarce. Several preceding studies have highlighted the importance of GCC as an indicator in diverse retinal diseases. There exists a noteworthy correlation between GCC and VA in conditions such as diabetic macular edema [12], retinal vein occlusion [16], and Behçet’s disease [14]. Hence, this study endeavored to identify GCC thickness and any GCC changes in acute CSC, investigating whether these could influence final VA. However, in the case of acute CSC, with the presence of SRF in the outer retina, it is improbable that this fluid could traverse multiple retinal layers to impact the GCC thickness. In addition, GCC thickness, including RNFL, GCL, and IPL in the presence of SRF did not correlate with visual prognosis following SRF absorption. Despite significant improvement in VA, no significant changes were observed in GCC thickness over the six-month follow-up period. These findings suggest that the VA of patients with resolved acute CSC is likely influenced more by abnormalities in the outer retina than changes in the Inner retina [2,17]. 

Our findings suggest that the thickness of the GCC in the inner retina is unaffected in acute CSC when SRF resolves before and after performing focal laser photocoagulation. Despite the absence of a consensus about the most accepted management modality for CSC, many approaches including observation, anti-vascular endothelial growth factor (VEGF) [18,19,20], photodynamic therapy [19,21,22], and laser therapy [19,23], have been proposed. Among them, traditional focal laser photocoagulation initiates focal coagulation at the level of the RPE within areas confirmed to be focal leakage points by fluorescein angiography [2,18,24]. It has been proposed that the focal laser injury instigates the recruitment of regular RPE cells or directly stimulates the RPE pumping function around the treated area, although the exact mechanism by which SRF resolution occurs following focal laser treatment remains undefined [2,19]. It appears that the laser acts upon the outer retina or choroid without significantly affecting the GCC in the inner retina, but further long-term follow-ups over 6 months are necessary to determine any changes in the GCC. 

Discrepancies exist in the literature regarding the impact of acute CSC on GCC thickness (Table 6). For instance, Demirok et al.’s cross-sectional study (n = 16) found a significantly lesser GCL-IPL complex thickness in acute CSC patients compared to a normal group using Cirrus OCT [6]. Nam et al. reported in their study (n = 30) on acute CSC patients that GCL-IPL thickness was less than in the contralateral eye when SRF was present but normalized once SRF was reabsorbed, yielding no differences from the contralateral eye [9]. The authors proposed that the segmentation error may contribute to the thinning of the GCL-IPL in the presence of SRF using Cirrus OCT [9]. During auto segmentation with the Cirrus OCT device in the presence of SRF, precise detection of the outer boundary of either the RNFL or IPL proved challenging, leading to an underestimation of the GCL-IPL thickness [9]. Jaisankar et al. reported a decrease in RNFL and GCL thickness (with no information about IPL) as SRF diminished in acute CSC [4]. However, their sample size was small (n = 7), and their study cohort included both those who underwent laser treatment (n = 3) and those who were merely observed (n = 4), and they did not elaborate on the reasons for the reduction in GCL and RNFL thickness. Two previous studies align with our findings [7,8]. Gawecki et al. [8] reported no significant differences in GCL-IPL thickness between acute CSC patients (n = 13) and a normal control group, while they noted a reduced GCL-IPL thickness in chronic CSC patients compared to controls. They surmised that chronic CSC patients experience loss of both the outer retinal layers and GCL, leading to significant visual impairment [8]. Likewise, Han et al. [7] examined 34 eyes with spontaneously resolved acute CSC, noting significant thinning of the outer retinal layer but an unaffected GCC layer.

Furthermore, it is crucial to recognize that the GCC measurements obtained in our study represent average values across a larger retinal area. This averaging may give an overall impression that acute CSC does not seriously affect the GCC. However, it is important to consider the possibility of minor, localized GCC damage, particularly in areas directly treated with laser. Although our study did not detect significant changes in GCC thickness on a broad scale, this does not completely rule out the presence of subtle GCC alterations in the focal areas of laser application. The potential for minor GCC damage in these localized spots, though not evident in our average measurements, underscores the need for more refined and localized assessment methods in future research to fully capture these subtle changes.

This study’s main limitation is its retrospective nature and small sample size. However, our findings provide consistent data on GCC thickness changes as all the participants that underwent focal laser treatment. Furthermore, we not only compared results with healthy control eyes but also with fellow eyes, reducing potential bias. Second, we investigated GCC changes over a short follow-up period of six months, which prevented us from evaluating long-term changes in GCC thickness. Even though no changes in GCC were observed over this short period, it is possible that decreases in GCC might be seen over more extended periods exceeding a year. Consequently, future research involving longer follow-ups is warranted. Finally, in our study, the only factor significantly associated with the final VA was the baseline VA. Since focal laser photocoagulation is a procedure that necessitates specialized skill, the characteristics of the physicians conducting focal laser photocoagulation might also influence the final VA. However, in this research, all focal laser photocoagulation procedures were conducted by a single physician (DDH), and SRF improved in all patients following focal laser photocoagulation, rendering it impossible to analyze the impact of physician-related factors.

This research is the first to assess GCC changes, including both RNFL, GCL, and IPL pre- and post-resolution of SRF in resolved acute CSC patients who underwent focal laser photocoagulation. During the first six months after focal laser photocoagulation, there were no significant changes in GCC thickness, and no notable association between the final VA and GCC thickness. The focal laser photocoagulation appears to have no significant effect on GCC thickness, and further studies with larger sample sizes are needed to track long-term GCC changes post-laser treatment.

## Figures and Tables

**Table 1:** Patient demographics and characteristics.

	CSC Group	Controls	*p*-Value
Patients (n)	30	45	
Age (years)	54.21 ± 9.72	54.51 ± 7.96	0.883 ^a^
Sex			0.377 ^b^
Male	21	27	
Female	9	18	
BCVA (logMAR)	0.20 ± 0.14	0.08 ± 0.15	**0.005**
IOP (mmHg)	14.60 ± 2.57	15.83 ± 2.85	0.101
CMT (µm)	423.90 ± 118.62	270.38 ± 24.28	<**0.001**

Values are presented as n or mean ± standard deviation. Bold font indicates statistically significant values (*p*-value < 0.05). OD = right eye; OS = left eye; BCVA = best-corrected visual acuity; IOP = intraocular pressure; CMT = central macula thickness; ^a^ *p*-value derived from the independent *t*-test; ^b^ *p*-values were derived from the Pearson’s Chi-square test.

**Table 2:** Comparison of the ganglion cell complex thickness between the central serous chorioretinopathy, fellow, and normal control eyes at baseline.

	CSC Eye	Fellow Eye	Control Eye	*p* ^a^	*p* ^b^	*p* ^c^
GCC	104.83 ± 10.82	105.18 ± 8.35	106.20 ± 6.41	0.155	0.494	0.566
RNFL	33.01 ± 4.85	32.95 ± 4.06	33.90 ± 3.61	0.159	0.366	0.310
GCL	39.56 ± 4.40	39.83 ± 3.39	39.88 ± 2.33	0.594	0.722	0.948
IPL	32.26 ± 3.04	32.40 ± 2.68	32.42 ± 2.20	0.863	0.799	0.975

Values are presented as mean ± standard deviation (µm). CSC= central serous chorioretinopathy; GCC = ganglion cell complex; RNFL = macular retinal nerve fiber layer; GCL = ganglion cell layer; IPL = inner plexiform layer; ^a^ Comparison between the CSC and fellows eyes at initial visit. *p*-values derived from the paired *t*-test; ^b^ Comparison between the CSC and normal control eyes at initial visit. *p*-value derived from the independent samples *t*-test; ^c^ Comparison between the fellow and normal control eyes at initial follow up. *p*-values derived from the independent samples *t*-test.

**Table 3:** Longitudinal changes in the ganglion cell complex thickness in central serous chorioretinopathy eye.

	Baseline	1 Month	3 Months	6 Months	*p*-Value ^a^
GCC	104.83 ± 10.82	105.20 ± 11.16	103.66 ± 12.71	105.16 ± 8.79	0.962
*p*-value ^b^		0.304	0.208	0.310	
RNFL	33.01 ± 4.85 (0.93 ± 0.14)	32.92 ± 4.81 (0.93 ± 0.14)	31.91 ± 5.44 (0.90 ± 0.15)	32.56 ± 3.95 (0.92 ± 0.11)	0.854
*p*-value ^b^		0.750	0.090	0.285	
GCL	39.56 ± 4.40 (1.12 ± 0.12)	39.59 ± 4.41 (1.12 ± 0.12)	39.32 ± 4.83 (1.11 ± 0.14)	39.71 ± 3.66 (1.12 ± 0.10)	0.993
*p*-value ^b^		0.889	0.331	0.064	
IPL	32.26 ± 3.04 (0.91 ± 0.09)	32.69 ± 3.21 (0.92 ± 0.09)	32.43 ± 3.57 (0.92 ± 0.10)	32.89 ± 2.58 (0.93 ± 0.07)	0.902
*p*-value ^b^		0.084	0.204	0.354	

Values are presented as mean ± standard deviation (µm). Values in parentheses represent the volume of each layer of the macula, expressed as mean ± standard deviation (mm^3^). GCC = ganglion cell complex; RNFL = macular retinal nerve fiber layer; GCL = ganglion cell layer; IPL = inner plexiform layer; ^a^ one-way ANOVA test; ^b^ Comparison between the baseline values and values at each time point. *p*-values derived from the paired *t*-test.

**Table 4:** Longitudinal changes in the ganglion cell complex thickness in the ETDRS sector with leakage in central serous chorioretinopathy eye.

	Baseline	1 Month	3 Months	6 Months	*p*-Value ^a^
GCC	109.70 ± 18.93	110.97 ± 19.13	108.95 ± 21.10	109.50 ± 17.14	0.984
*p*-Value ^b^		0.053	0.903	0.417	

Values are presented as mean ± standard deviation (µm). GCC = ganglion cell complex; ^a^ one-way ANOVA test; ^b^ Comparison between the baseline values and values at each time point. *p*-values derived from the paired *t*-test.

**Table 5:** Final visual acuity and associated factors through multiple regression analysis.

	Final VA	
	Standardized Coefficients Beta	*p*-Value
Age	−0.303	0.320
Sex	0.443	0.053
Baseline VA	0.697	**0.017**
Baseline RNFL	−0.961	0.131
Baseline GCL	1.866	0.168
Baseline IPL	1.120	0.168
RNFL at 6 months	1.578	0.072
GCL at 6 months	−2.671	0.106
IPL at 6 months	−0.331	0.561

Bold font indicates statistically significant values (*p*-value < 0.05). VA = visual acuity; RNFL = macular retinal nerve fiber layer; GCL = ganglion cell layer; IPL = inner plexiform layer.

**Table 6:** Summary table for describing prior studies investigating the impact of acute CSC on GCC thickness using OCT images.

Authors	Subjects (Number)	OCT Machine	Study Focus	Key Findings
Demirok et al. [6]	16	Cirrus SD-OCT	GCL-IPL thickness in acute CSC vs. normal group	Lesser GCL-IPL thickness in acute CSC
Nam et al. [9]	30	Cirrus SD-OCT	GCL-IPL thickness change with/without SRF in acute CSC	GCL-IPL thickness less with SRF, normalized after SRF reabsorption
Jaisankar et al. [4]	7	Topcon SS-OCT	RNFL and GCL thickness change with SRF in acute CSC	Decrease in RNFL and GCL thickness as SRF diminished
Gawecki et al. [8]	13	REVO NX SD-OCT	GCL-IPL thickness in acute and chronic CSC vs. controls	No significant difference in acute CSC but reduced GCL-IPL thickness in chronic CSC
Han et al. [7]	34	Topcon SS-OCT	Outer retinal layer and GCC layer thickness in resolved acute CSC	Thinning of outer retinal layer but unaffected GCC in resolved acute CSC
Our study	30	Spectralis SD-OCT	GCC, RNFL, GCL, IPL thickness	No significant change

CSC = central serous chorioretinopathy; OCT = optical coherence tomography; SD-OCT = spectral-domain OCT; SS-OCT = swept-source OCT; GCC = ganglion cell complex; RNFL = macular retinal nerve fiber layer; GCL = ganglion cell layer; IPL = inner plexiform layer.

## Data Availability

The data are not available for public access because of patient privacy concerns but are available from the corresponding author upon reasonable request.
